# Experimental Investigation of the Size Effect on Roller-Compacted Hydraulic Asphalt Concrete under Different Strain Rates of Loading

**DOI:** 10.3390/ma17020353

**Published:** 2024-01-10

**Authors:** Xiao Meng, Yunhe Liu, Zhiyuan Ning, Jing Dong, Gang Liang

**Affiliations:** State Key Laboratory of Eco-hydraulics in Northwest Arid Region of China, Xi’an University of Technology, Xi’an 710048, China; mxlucky0928@163.com (X.M.); ningzhiyuan1108@163.com (Z.N.); 1190411033@stu.xaut.edu.cn (J.D.); nieyanzhizhang@163.com (G.L.)

**Keywords:** hydraulic asphalt concrete, failure modes, strain rate effect, size effect, dynamic size effect law

## Abstract

**Highlights:**

(1) Failure modes of RCHAC under the coupling effect of the strain rate effect and the size effect were analyzed.
(2) Dynamic mechanical properties under different size and strain rates were presented.
(3) The effect of strain energy on failure modes was analyzed.
(4) A modified dynamic size effect law was proposed.

**Abstract:**

Asphalt concrete is widely used in hydraulic structure facilities as an impermeable structure in alpine cold regions, and its dynamic mechanical properties are influenced by the strain rate and specimen size. However, the specimen size has an important effect on mechanical properties; few systematic studies have investigated on the size effect of hydraulic asphalt concrete (HAC) under dynamic or static loading rates. In the present study, four sizes of cylindrical roller-compacted hydraulic asphalt concrete (RCHAC) specimens with heights of 50 mm, 100 mm, 150 mm, and 200 mm were prepared and tested under different loading rates ranging from 10^−5^ s^−1^ to 10^−2^ s^−1^ to investigate the size effects of mechanical properties and failure modes at the temperature of 5 °C. The effect of strain rate on the size effects of the compressive strength and the elastic modulus of RCHAC have also been explored. These tests indicate that when the specimen size increases, the compressive strength and failure degree decrease, while the elastic modulus increases. When the height increases from 50 mm to 200 mm, the compressive strength at different strain rates decreased by more than 50%. Furthermore, the elastic modulus increased by about 211.8% from 0.51 GPa to 1.59 GPa at a strain rate of 10^−5^ s^−1^, and increased by 150% from 5.08 GPa to 12.71 GPa at a strain rate of 10^−2^ s^−1^. As the strain rate increases, the variation trends with the size of the compressive strength, elastic modulus, and failure degree are distinctly intensified. A modified dynamic size effect law, which incorporates both the specimen size and strain rate, is proposed and verified to illustrate the dynamic size effect for the RCHAC under different loading rates.

## 1. Introduction

Since the first roller-compacted hydraulic asphalt concrete (RCHAC) core embankment was built in Germany in 1962, this extremely competitive type of dam has been widely used in many countries due to its advantages of good seepage prevention, earthquake resistance, and deformation adaptability [1]. The damage to the asphalt core wall will greatly reduce its bearing capacity and impermeability, which will seriously threaten the safety performance of the whole dam. In many countries, lots of hydraulic structures are built in the alpine cold region; with low temperatures and frequent earthquakes, the dynamic mechanical safety performance of the RCHAC core wall has always been the concern of researchers. There have been many efforts to study the mechanical properties and failure mechanisms of RCHAC [2,3,4,5,6]; the majority of these, however, ignored the effect that size has on mechanical properties, while many studies showed that the size of the specimen has a significant effect on the mechanical properties of heterogeneous concrete-like materials [7,8,9,10,11]. Therefore, another noteworthy aspect is the influence of the specimen size on the mechanical properties and failure mechanisms in the mechanical research of HAC.

Considering the influence that size has on the mechanical properties of asphalt concrete, Wang [12], through bending tests of asphalt concrete of different sizes, concluded that the deformation of large-size specimens was larger, while the strength was slightly lower, initially confirming that the mechanical properties of HAC were affected by size. Kim [13] carried out static tensile tests and discrete element numerical simulations on disc specimens of asphalt concrete of differing sizes, further demonstrating that the dependence of the tensile strength of asphalt concrete on size is similar to that of ordinary cement concrete; with the increase in specimen size, the tensile strength decreases while the fracture energy increases. In addition, Liu [14] and Hagighat [15] also conducted numerical simulations and mechanical experimental studies on the size effect of asphalt concrete. In general, there are relatively few studies on the size effect of the mechanical properties of RCHAC. Moreover, due to the limitations of test conditions and other factors, there is a lack of data on the effect size has on the dynamic mechanical properties of asphalt concrete, which needs to be further improved.

RCHAC is also a rate-sensitive material [12,16]. The mechanical response under dynamic loads, such as explosions or earthquakes, is significantly different from that under static loads, which is the strain rate effect behavior. Nakumara [17] reported that the strain rate has a significant effect on the dynamic tensile strain failure of asphalt concrete. Wang [5] conducted shear tests on asphalt concrete and concluded that shear modulus, shear strength, and cohesion increased with an increase in strain rate. Ning [18] carried out compressive performance tests of RCHAC under dynamic loads with different strain rates, and their results showed that the strain rate significantly influenced the mechanical properties and failure modes of RCHAC. Therefore, it is necessary to attribute more attention to the effect of the strain rate when studying the dynamic mechanical properties of RCHAC.

In summary, both the size effect and the strain rate effect should be considered in research. However, there is relatively little research on the size effect of RCHAC under dynamic loads. Chen [19] and Albayati [20] reported that HAC has elastic–brittle mechanics and characteristics in low-temperature environments. Therefore, for the dynamic size effect of RCHAC in alpine cold regions, previous studies on the size effect of concrete-like materials carried out by other researchers can be used as references. Krauthammer [21] and Elfahal [22] performed experimental research on the cylindrical concrete specimen with different dimensions under loading rates ranging from 0.014 s^−1^ to 3.03 s^−1^. They found that the dynamic compressive strength of the cylindrical concrete decreases with the increase in the specimen’s size, presenting a significant size effect. Liang [23] tested the uniaxial compression of rock specimens with different ratios of height to diameter (i.e., *L/D* = 0.5, 0.75, 1, and 1.25) under the strain rates ranging from 10^−5^ s^−1^ to 10^−2^ s^−1^. They found that with the increase in the specimen’s height, the strength and peak strain decrease, the elastic modulus increases, the degree of failure declines, and the dynamic mechanical response is less sensitive towards the strain rate. Jin [24] established random aggregate models of concrete with different sizes under dynamic compression loading at different strain rates ranging from 10^−5^ s^−1^ to 100 s^−1^. They found that the influence of the dimension on the compressive strength under static and dynamic loading was obviously different, and that the dynamic and static unified size effect of concrete compressive strength was established based on the influence mechanisms of the strain rate effect and the size effect.

Based on the above research method for the dynamic size effect, uniaxial compression experiments of RCHAC with different sizes under different strain rates were carried out to study the combined effects of the strain rate effect and the size effect on the mechanical properties and failure modes of RCHAC specimens with different sizes under dynamic loading rates from 10^−5^ s^−1^ to 10^−2^ s^−1^ (i.e., an earthquake) at a low temperature (5 °C) and establish a dynamic size effect law for mechanical properties based on the influencing mechanism of the strain rate effect on dynamic properties and the size effect. This research shows the need for a reasonable transition between scaled mechanical tests in the laboratory and the actual engineering situation, provides data and theoretical support for the study of the dynamic mechanical properties of hydraulic asphalt concrete, and promotes the development of asphalt concrete anti-seepage materials.

## 2. Materials and Methods

### 2.1. Specimen Preparation

**Figure 1:**
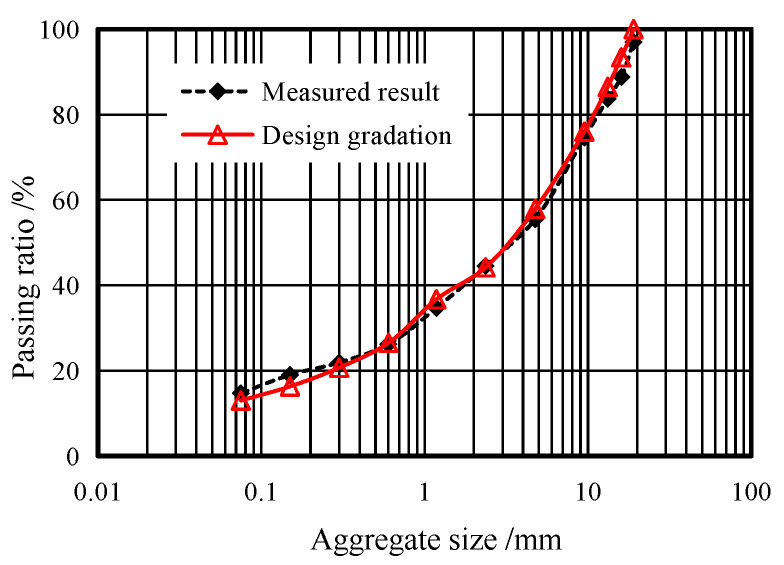
Aggregate gradation curve of RCHAC.

**Figure 2:**
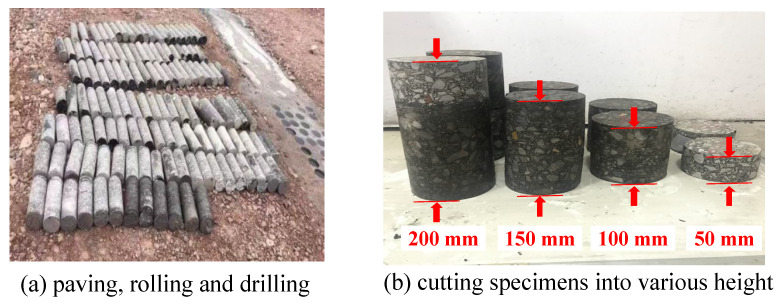
Specimen preparation.

In this experiment, the asphalt core specimen was prepared at a roller-compacted hydraulic asphalt concrete (RCHAC) core dam construction site in Northwestern China. The grading index of the asphalt mixture was 0.4, and the aggregate gradation curve is shown in Figure 1. The aggregate was crushed limestone. The added filler (<0.075 mm) was limestone powder and accounted for 13% of mineral mass. The bitumen was of grade B90, and the bitumen content was 7% of the mineral weight. The porosity was about 1.7% to 2.1%. The asphalt and aggregates were mixed in a mixing plant in accordance with the above proportions, and the discharging temperature was 165 °C. The asphalt mixture was paved and pre-rolled into a test section (Figure 2a) with a paver equipped with a vibrating iron compactor, and the pre-compaction coefficient was 86%. Then, when rolling the asphalt concrete, the initial rolling temperature was not less than 150 °C, and the final rolling temperature was not less than 110 °C. After curing, asphalt concrete cores with diameters of 100 mm were drilled and cut into four differently sized cylinders with heights of 50 mm, 100 mm, 150 mm, and 200 mm (Figure 2b). The ratios of height to diameter (*L/D*) of the specimens were 0.5, 1, 1.5, and 2. According to the Chinese standard DL/T 5362-2006 [25], the specimen with the height of 100 mm was used as the standard specimen for the uniaxial compression test of RCHAC.

### 2.2. Experimental Methods

**Figure 3:**
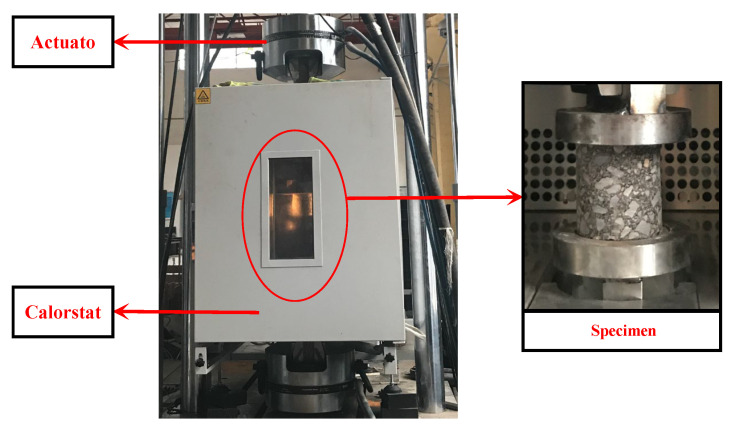
The dynamic loading system and calorstat.

Dynamic compression tests were carried out on three specimens under different strain rates in each group using the MTS dynamic fatigue test system (Figure 3). The test temperature was set at 5 °C, which was determined according to the temperature in the asphalt core located in the central region of embankment dams in the alpine cold region [2]. Before loading, the specimens were kept at the constant temperature at 5 °C for at least 48 h. During the whole experiment, the test temperature was kept constant using a calorstat (Figure 3). In order to accurately reveal the size dependence of asphalt concrete, the constant strain rate method was adopted in this uniaxial compression experiment. The strain rates of these tests ranged from a quasi-static strain rate of 10^−5^ s^−1^ to the dynamic strain rates of 10^−4^ s^−1^, 10^−3^ s^−1^, and 10^−2^ s^−1^ (i.e., an earthquake) [26]. This test was set up in four groups according to the ratio of height to diameter: SE-1, SE-2, SE-3, and SE-4 (i.e., *L/D* = 0.5, 1, 1.5, and 2). The real-time force and displacement data of the test were acquired automatically through the acquisition module of the MTS system. The average value of the tested results of three specimens in each group was recorded if the difference between the average value and the maximum or minimum value of the tested result was less than 15%.

## 3. Results and Analysis

### 3.1. Compressive Strength

**Figure 4:**
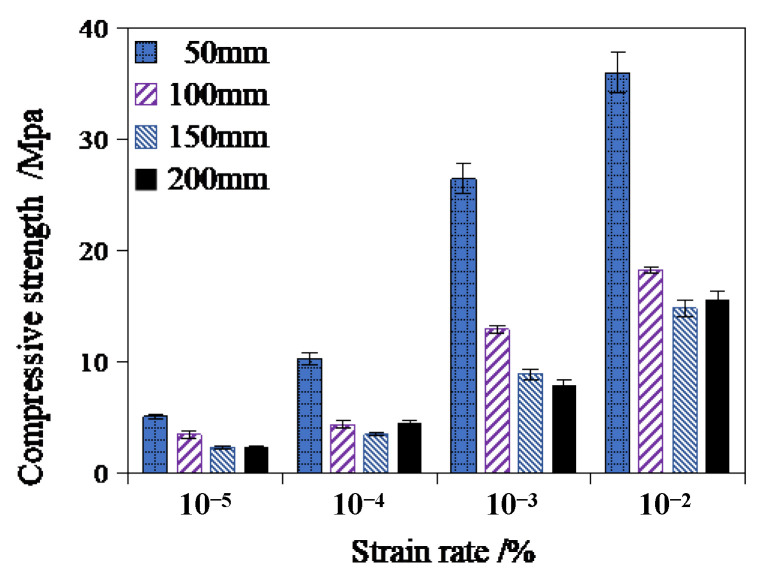
Test results of RCHAC compressive strength under different strain rates.

Table 1 and Figure 4 show the average compressive strength of RCHAC specimens of different sizes under different loading rates. It can be seen that the compressive strength of the RCHAC decreases non-linearly with the increase in height, and the decrease trend is slower with the increase in specimen height. When the specimen height increases from 50 mm to 200 mm, the compressive strength decreases from 5.01 MPa to 2.30 MPa at the strain rate of 10^−5^ s^−1^, and the compressive strength decreases from 35.98 MPa to 15.59 MPa at the strain rate of 10^−2^ s^−1^. As shown in Figure 4, when the strain rate increases, the compressive strength decreases more significantly with the increase in size.

Meanwhile, the compressive strength of the RCHAC increases with the increase in strain rate. When the strain rate increases from 10^−5^ s^−1^ to 10^−2^ s^−1^, the compressive strength of the specimen at the height of 50 mm increases from 5.01 MPa to 35.98 MPa, with an increase of 618.16%; the compressive strength of the 200-mm specimen increases by 577.83%, from 2.30 MPa to 15.59 MPa, showing an obvious strain rate enhancement effect. When the temperature of the experiments is above 0 °C, the viscous stress of asphalt increases rapidly with the increase in the strain rate, so the compressive strength of asphalt concrete is greatly enhanced with the increase in strain rate [27]. As shown in Figure 4, the compressive strength and strain rate of the RCHAC show a positive correlation trend, and this overall trend gradually weakens with the increase in the height of the specimen. Therefore, the variation law of the compressive strength of RCHAC of different sizes under dynamic load is the result of the coupling influence of the size effect and the strain rate effect. This is consistent with the research results on the size effect of the dynamic mechanical properties of rocks and concrete conducted by Mattia [28] and Milad [29].

### 3.2. Elastic Modulus

**Figure 5:**
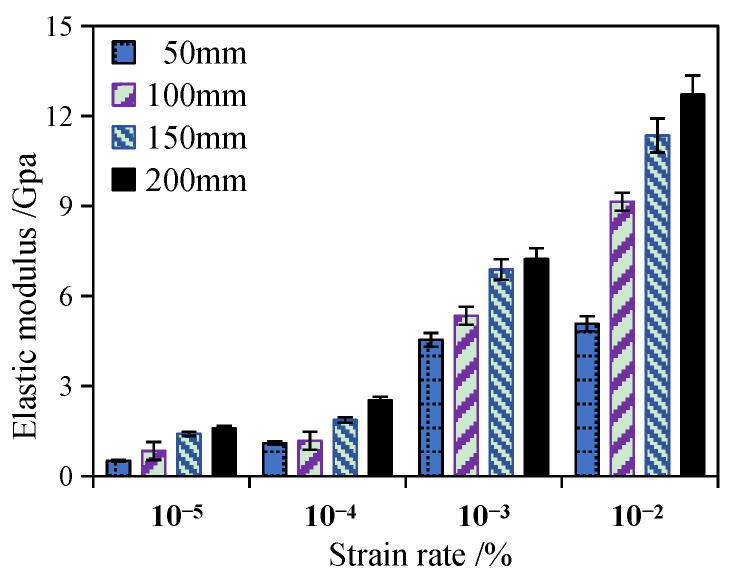
Test results of the elastic modulus of RCHAC under different strain rates.

It can be seen from Table 2 and Figure 5 that when the height of the specimen increases from 50 mm to 200 mm and the strain rate is 10^−5^ s^−1^, the elastic modulus increases from 0.51 GPa to 1.59 GPa. When the strain rate is 10^−2^ s^−1^, the elastic modulus of the specimen increases from 5.08 GPa to 12.71 GPa, which means that the elastic modulus of the specimen has a size effect that gradually increases with the increase in its height. Additionally, Figure 5 shows that when the strain rate increases, the positive correlation between the elastic modulus and the specimen height becomes more obvious.

For the same specimen size, with the increase in strain rate, the elastic modulus also presents a non-linear increase, and the larger the strain rate, the more obvious the change trend. As can be seen from Table 2, as the strain rate increases from 10^−5^ s^−1^ to 10^−2^ s^−1^, the elastic modulus of the specimen at the height of 50 mm increases from 0.51 GPa to 5.08 GPa, with an increase of 896.08%. The elastic modulus of the specimen at the height of 200 mm increased by 699.37% from 1.59 GPa to 12.71 GPa. The variation law of elastic modulus with strain rate shows a similar enhancement effect to the research results of Ning [18,30]. It is also shown that the strain rate effect of the elastic modulus of RCHAC decreases with an increase in dimension height. It can be known that the variation law of the elastic modulus of RCHAC is the result of the coupling effect between the size effect and the strain rate effect.

### 3.3. Failure Modes

**Figure 6:**
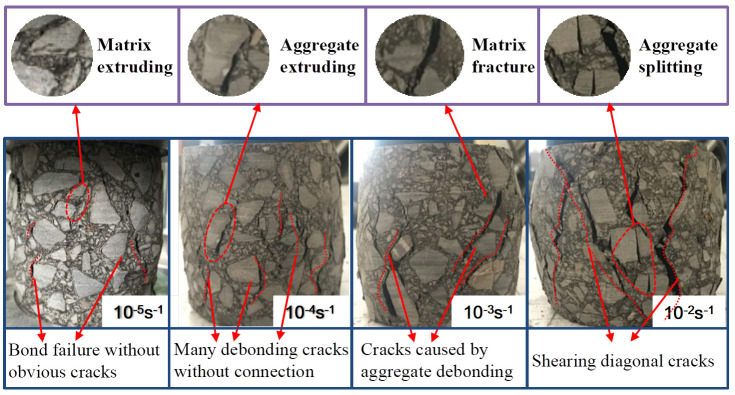
Failure modes of 100-mm-high specimens at different strain rates.

Figure 6 shows the failure mode of specimens with a height of 100 mm under different strain rates. It can be seen that the failure mode of specimens is greatly affected by the strain rate, which is also reflected in the study by Tekalur [27]. As can be seen from Figure 6, when the strain rate is 10^−5^ s^−1^, there is no obvious damage to the aggregate on the surface of the specimen, only the small cracks caused by the debonding of the aggregate but no penetrating cracks. Due to the slow loading rate and relatively uniform distribution of stress inside the specimen, with the increase in stress, cracks first appear in the weak area, such as the interface between the aggregate and asphalt matrix, and then develop along the weak area around the aggregate. Meanwhile, the coarse aggregates extrude each other to gradually extrude the bonding material, the asphalt matrix. Therefore, the failure pattern of the specimen at a low strain rate is bond failure, which is mainly caused by the extrusion of the asphalt matrix and the debonding of the aggregate and matrix. With the increase in strain rate, some phenomena, such as asphalt matrix fracture and extrusion, coarse aggregate crushing, and aggregate vertical fracture, appear on the surface of the specimen. When the strain rate is 10^−2^ s^−1^, multiple cracks on the surface of the specimen gradually connect through others, and multiple oblique cracks appear across the surface of the specimen. The above phenomenon occurs when the loading rate becomes faster and the internal stress distribution of RCHAC is more uniform. Therefore, although the weakest area has not reached the strength limit, many areas have reached the strength limit and cracks have occurred, and the generation and development of cracks is faster. The stress does not release along the shortest path of the weak area; it even releases through part of the aggregate to form cracks, and these cracks intersect each other to form multiple penetrating diagonal cracks.

**Figure 7:**
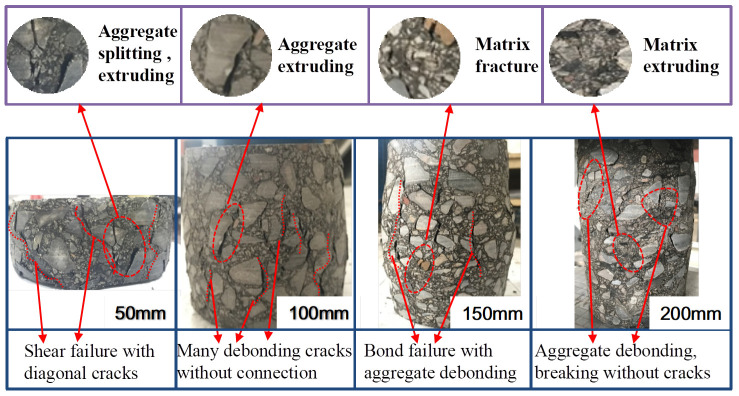
Failure modes of specimens of different heights at a 10^−3^ s^−1^ strain rate.

Figure 7 shows the failure mode of specimens with different heights at a strain rate of 10^−3^ s^−1^. It can be seen that under the same loading rate, the failure mode of the specimen has a significant level of sensitivity to its size. When the height is 50 mm, part of the aggregate is fractured, and some of the aggregate is extruded out of the surface, and there are clear diagonal cracks on the surface of the specimen. At this time, the failure mode is mainly shear failure. As the height is increased, the proportion of influence of the specimen internal defects on the performance of RCHAC increases, and the influence of the structural effect (i.e., including lateral restraint and end friction effects) on the material decreases. The diagonal cracks on the surface of the specimen are distinctly reduced, and the phenomenon of aggregate crushing and extrusion is also gradually reduced. When the height is increased to 200 mm, the aggregate is complete without exhibiting obvious damage, and there is not any through cracks on the specimen; at this time, the failure mode is the bond failure caused by the debonding of the asphalt matrix and aggregate. With the increase in height, the damage degree of the specimen decreases obviously.

It can be seen from Figure 6 and Figure 7 that the failure degree of the specimen decreases with the decrease in strain rate or the increase in specimen height. It shows that the failure process of the asphalt concrete specimen is not a single mode, and its failure mode is affected by the strain rate and specimen size.

### 3.4. Strain Energy Analysis of Failure Modes

According to the thermodynamics theory, the comprehensive effect of the energy conversion process is the driving factor of material failure. Assuming that the uniaxial compression test system is a closed system without heat exchange with the outside world, the total input strain energy, U , of the asphalt concrete specimen under uniaxial compression is as follows:

(1) $$U=U^{d}+U^{e}$$

where *U*^d^ is the dissipative strain energy, which is used to induce internal damage and plastic deformation of the specimen, and it also contains viscous strain energy due to the influence of asphalt concrete viscosity. *U*^e^ is elastic strain energy, and the release of elastic strain energy stored in the specimen is the internal cause of cracks and failure.

**Figure 8:**
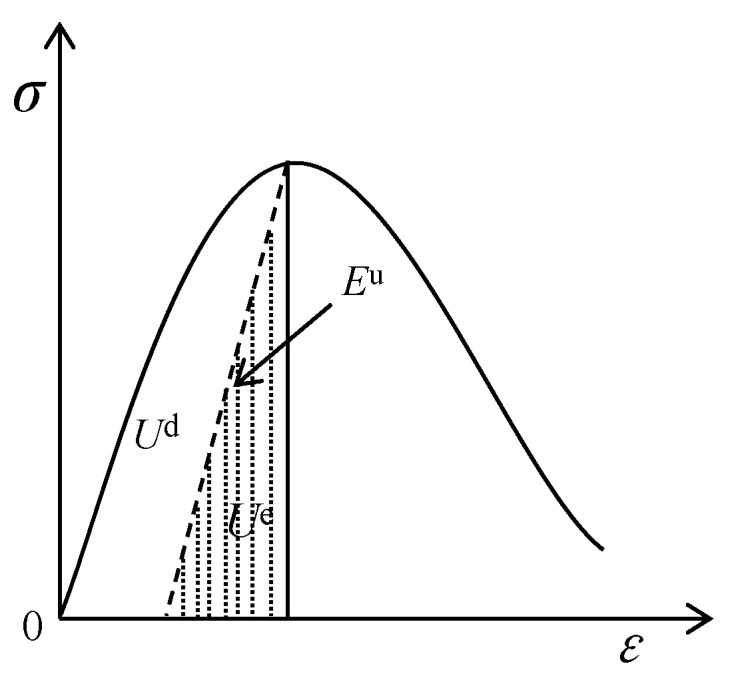
Dissipation strain energy and release strain energy under uniaxial compression.

**Figure 9:**
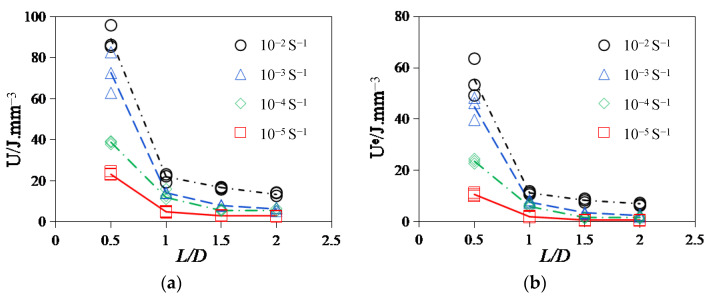

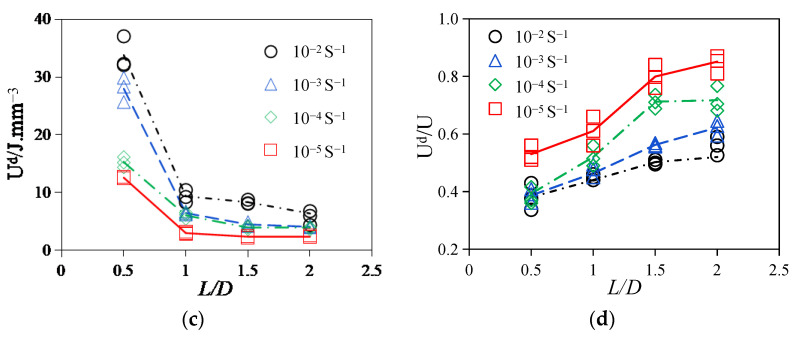
The size effect of various energies at different strain rates. (**a**) Total absorbed energy, *U*; (**b**) elastic strain energy, *U*^e^; (**c**) dissipated strain energy, *U*^d^; and (**d**) *U*^d^/*U*.

Figure 8 shows the relationship between the energies in the stress–strain curve under uniaxial compression. The area enclosed by the curve and unloading modulus in the figure is the dissipated strain energy, and the triangular shaded area is the elastic strain energy. It should be noted that since the unloading test was not carried out in this experiment, the initial elastic modulus was used for energy calculation. Then, the energy of each part was calculated using Equations (1)–(3), as shown in Figure 9a–c.

(2) $$U=\int_{0}^{\varepsilon_{p}}\sigma d\varepsilon$$

(3) $$U^{e}=\frac{1}{2}\cdot \sigma_{p}\cdot \varepsilon^{e}\approx \frac{1}{2E_{0}}\cdot \sigma_{p}^{2}$$

where *ε*_p_ is the peak strain; *σ*_p_ is the compressive strength; *ε*^e^ is the elastic strain; and E _0_ is the modulus of elasticity.

It can be seen from Figure 9 that the variations in total absorption energy, *U*, dissipated strain energy, *U*^d^, and elastic strain energy, *U*^e^, in the process of compressive strength of all sizes of specimens are as follows: the *U*, *U*^d^, and *U*^e^ of the specimens will increase with the increase in strain rates. Corresponding to the failure characteristics in Figure 6 and Figure 7, it can also be seen that due to the increase in *U* per unit volume, a large number of cracks will be generated due to higher energy absorption, and the development of cracks needs to consume more energy.

Hence, the larger the strain rate, the more cracks will be generated during the final failure of the specimen, and the more distinct the brittle failure characteristics [31]. However, when the specimen size increases, *U*, *U*^d^, and *U*^e^ all show a decreasing trend, and the amplitude of this decreasing trend becomes more obvious with the increase in strain rate. This is also consistent with the change rule of compressive strength in Figure 4, indicating that the strength of the specimen is related to the internal storage energy. The higher the value of *U*^e^, the more energy is released when the specimen is damaged, and the bigger the strength [32]. Moreover, as the specimen increases and the strain rate decreases, *U*^e^/*U* shows a gradually decreasing trend while *U*^d^/*U* shows an increasing trend, indicating that the proportion of elastic strain energy used to release decreases at this time, and the degree of brittle failure of the specimen also decreases. An increase in the proportion of *U*^d^ indicates that the damage dissipation energy increases, the viscous strain energy increases, the rate of stress softening slows down, and the number of cracks decreases during failure. Thus, Figure 9 shows that the sensitivity of the asphalt concrete failure mode to size increases with the strain rate [33]. The tendency of the dissipated energy of RCHAC to decrease with increasing size is more pronounced for larger strain rates, and the slope of the change curve is larger [34]. When the specimen is large and the strain rate is low, the plastic damage of the specimen is greater, and the ‘strength loss’ increases due to the increase in the viscosity work of the asphalt concrete. It should be noted that ‘loss of strength’ does not mean ‘holistic failure’ [35]. It is because the proportion of viscous dissipation energy of the specimen increases that the release of residual elastic strain energy after the damage is not enough to break through the surface strain energy of the specimen and generate large cracks, so the damage to the specimen increases but the degree of failure decreases.; that is to say, when the specimen size is large and the strain rate is small, there is no distinct crack and the degree of failure is low.

## 4. The Dynamic Size Effect Theory

### 4.1. The Size Effect on Mechanical Properties

With great efforts to understand the size effect of quasi-brittle materials, many researchers have recognized that the size effect of quasi-brittle materials under compression is essentially a matter of material science [8], which is closely related to the material’s aggregate particle size, shape, spatial distribution, pores, initial defects, and other micro- and meso-structural elements. Former researchers made a lot of explorations and published their corresponding important theories [35,36,37]. Currently, there are three main theories that describe the size effect: the size effect theory based on the weakest link and random strength theory, energy release, and multifractal theory. Among these, the size effect law based on the linear elastic fracture mechanics proposed by Bažant [26] has been proven to be able to describe the effect of the size effect on the properties of quasi-brittle materials. To further study the change law of the compressive strength and elastic modulus of RCHAC with height, the Bažant [8] size effect law is used to describe it:

(4) $$\sigma_{0}=\frac{B\cdot f_{t}}{\sqrt{1+\frac{D}{D_{0}}}}$$

where *D* denotes the structural size; herein, it is the diameter of the cylinder specimen. σ_0_ is the strength when the specimen size is *D*. f _t_ is the strength of the specimen at the height of 100 mm. *B*, *D*_0_ is the empirical parameter, which is obtained by fitting and used to determine the value range of *σ*_0_.

Based on the analysis of experimental data, Equation (4) can be modified to analyze the size effect of mechanical properties as follows:

(5) $$F_{c}=\frac{\beta_{c}\cdot F_{c,100}}{\sqrt{1+\frac{\gamma }{\gamma_{c}}}}$$

where *γ* is the height-to-diameter ratio of the cylindrical specimen, and the values in this experiment are 0.5, 1, 1.5, and 2. *F*_c_ is the mechanical parameter (e.g., compressive strength or elastic modulus) at any ratio of height to diameter. *F*_c,100_ is the experimental mechanical parameter of the 100-mm-height specimen. *β*_c_ and *γ*_c_ are the fitting parameters corresponding to different *F*_c_ values.

**Figure 10:**
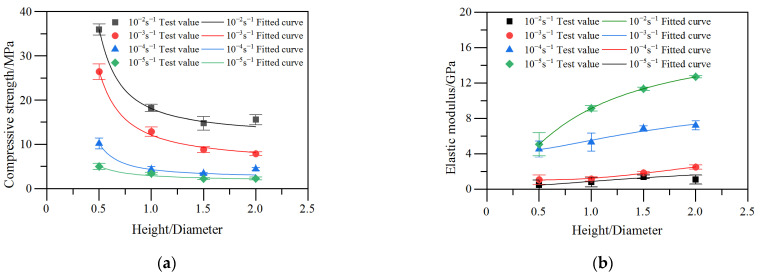
The experimental and theoretical values of the mechanical parameters of RCHAC of different sizes under different strain rates. (**a**) Compressive strength. (**b**) Elastic modulus.

Figure 10 shows the test values of compressive strength and the elastic modulus of RCHAC specimens under different strain rates, as well as the theoretical curves calculated using Equation (5). It is easy to understand from the slope changes of each curve in Figure 10 that with the increase in the height/diameter ratio of asphalt concrete specimens, the compressive strength tends to decrease while the elastic modulus tends to increase, and the trend of both tends to be flat. Therefore, the strain rate can enhance the dimensionality sensitivity of the asphalt concrete strength but suppress the dimensionality sensitivity of the elastic modulus [33,34]. Moreover, the strain rate can enhance the dimensionality sensitivity of the strength of RCHAC but suppress the dimensionality sensitivity of the elastic modulus.

In addition, as can be seen from the analysis in Figure 10, within the range of test loading rates, with the decrease in strain rate, the compressive strength and elastic modulus of specimens of the same size gradually decrease, while the peak strain gradually increases. As the strain rate further decreases, the variation trend of the size effect curve of the compressive strength and elastic modulus is more gradual.

As can be seen from Table 3, the mechanical parameters *β*_c_ and *γ*_c_ are closely related to the loading strain rate. Within the test conditions, with the increase in strain rate, each parameter presents a regular variation trend. It can be seen that Bažant’s [8] size effect law under medium and low strain rate conditions cannot accurately describe the size effect of RCHAC. Therefore, it is necessary to modify the Bažant size effect law, considering the effect of strain rate, so as to make a more comprehensive analysis of the compressive performance of RCHAC.

### 4.2. The Strain Rate Effect

The existing experimental results show that the dynamic mechanical properties of brittle or quasi-brittle materials, such as rocks and concrete, have a significant strain rate effect [26,38]. In this paper, by exploring the change law of compressive strength and the elastic modulus of RCHAC specimens under different strain rates (10^−5^ s^−1^~10^−4^ s^−1^), based on the research results of CEB-FIP [39] and Ning [40], the compressive dynamic increase factor (CDIF) about strain rate is introduced to show the strain rate effect of mechanical properties of RCHAC more intuitively and uniformly. CDIF (*F*_c_) is defined as the ratio of the mechanical properties under the condition of the dynamic strain rate to the values under the condition of the 10^−5^ s^−1^ strain rate. Through calculation and analysis, it was found that the compressive strength CDIF (*σ*_0_), the elastic modulus CDIF (*E*_0_), and the logarithm of strain rate of each group of specimens show a non-linear relationship. After analyzing the test data, Equation (6) was used for fitting analysis as follows:

(6) $$\mathrm{CDIF}\left(F_{c}\right)=\frac{P_{d}}{P_{s}}=exp\left[\alpha \cdot lg\left(\frac{{\dot{\varepsilon }}_{d}}{{\dot{\varepsilon }}_{s}}\right)\right]$$

where *F*_c_ is the mechanical parameter; *P*_d_ and *P*_s_ are the mechanical parameters under dynamic loading and quasi-static loading, respectively; ${\dot{\varepsilon }}_{d}$ is the dynamic strain rate; ${\dot{\varepsilon }}_{s}$ represents the quasi-static strain rate 10^−5^ s^−1^; and *α* is the material parameter obtained through fitting. The regression relationship between the compressive strength CDIF (*σ*_0_), the elastic modulus CDIF (*E*_0_), and the strain rate were obtained through fitting the above data.

The specific forms of Equation (1) for each mechanical property are as follows. The fitting parameters are shown in Table 4.

(7) $$\mathrm{CDIF}\left(\sigma \right)=\frac{\sigma_{d}}{\sigma_{s}}=exp\left[\alpha_{1}\cdot lg\left(\frac{{\dot{\varepsilon }}_{d}}{{\dot{\varepsilon }}_{s}}\right)\right]$$

(8) $$\mathrm{CDIF}\left(E\right)=\frac{E_{d}}{E_{s}}=exp\left[\alpha_{2}\cdot lg\left(\frac{{\dot{\varepsilon }}_{d}}{{\dot{\varepsilon }}_{s}}\right)\right]$$

**Figure 11:**
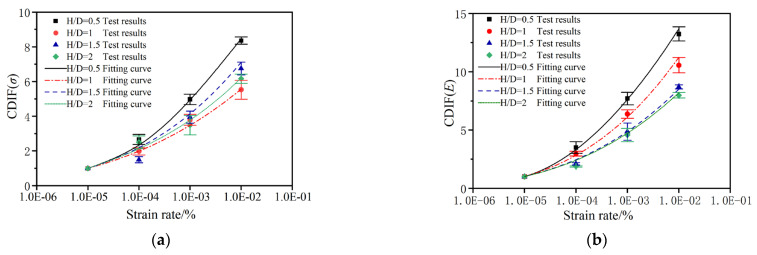
Trend regression curve of the CDIF with the strain rate of RCHAC under different heights. (**a**) The CDIF of compressive strength. (**b**) The CDIF of elastic modulus.

Figure 11a,b show the variation curve of CDIF values calculated from the test data and the fitting result on the logarithm of strain rate. It can be seen from the distribution of CDIF values and the trend of the fitting curve that the compressive strength and elastic modulus of RCHAC specimens with different heights are significantly affected by the strain rate. As mentioned above, the CDIF values represent the increase or decrease in mechanical properties at dynamic loading rates and quasi-static loading rates. It can be seen from the calculation results and Figure 11 that under the condition of the dynamic loading rate, the compressive strength CDIF and elastic modulus CDIF are both greater than one, which indicates that the compressive strength and elastic modulus have significant strain rate enhancement effects. Meanwhile, the amplitude of compressive strength enhancement also increases significantly with the increase in strain rate.

In addition, the dependence of CDIF values on the strain rate varies with different sizes; that is to say, the compressive strength and elastic modulus corresponding to the variation amplitude of the strain rate show size effects. As shown in Figure 11, the change rate of compressive strength and elastic modulus is the largest when the height is 50 mm. With the increase in the height, the change rate of each parameter decreases, and the strain rate effect decreases, which indicates that the strain rate effect of RCHAC has a significant size sensitivity in the strain rate range from 10^−5^ s^−1^ to 10^−2^ s^−1^.

### 4.3. The Dynamic Size Effect Model

Considering the influence of the strain rate on the mechanical properties and size effect of the specimen, the influence coefficient of the strain rate, $\;\varphi_{\dot{\varepsilon }}$ , i.e., Equation (6), is introduced to modify Equation (5) to obtain Equation (9). Equation (9) is the theoretical model of the size effect of RCHAC considering the dependence of the strain rate under medium and low strain rates:

(9) $$F_{c}=\frac{\beta_{c}\cdot F_{c,100}^{{\dot{\varepsilon }}_{s}}}{\sqrt{1+\frac{\gamma }{\gamma_{c}}}}\cdot \varphi_{\dot{\varepsilon }}$$

which is

(10) $$F_{c}=\frac{\beta_{c}\cdot F_{c,100}^{{\dot{\varepsilon }}_{s}}}{\sqrt{1+\frac{\gamma }{\gamma_{c}}}}\cdot exp\left(lg\frac{{\dot{\varepsilon }}_{d}}{{\dot{\varepsilon }}_{s}}\right)$$

where $F_{c,100}^{{\dot{\varepsilon }}_{s}}$ denotes the mechanical index of asphalt concrete specimens with a height of 100 mm and under a quasi-static loading rate (10^−5^ s^−1^).

Equation (10) can be rearranged as follows:

(11) $$Z=z_{0}\cdot \frac{\beta_{c}}{\sqrt{1+\frac{x}{\gamma_{c}}}}\cdot exp\left[\alpha \cdot \left(y+5\right)\right]$$

where *x* = *γ*, *y* = lg( ${\dot{\varepsilon }}_{d}$ ), *Z* = *F*_c_, *z*_0_ = $F_{c,100}^{{\dot{\varepsilon }}_{s}}$ , and ${\dot{\varepsilon }}_{s}$ = 10^−5^ s^−1^.

In order to further verify this theoretical model, the theoretical size effect model was used to calculate the compressive strength and elastic modulus under the experimental conditions and compared with the experimental results. In this theoretical model, *z*_0_ is taken as the mechanical properties of the 100-mm-high specimens under the quasi-static loading rate of 10^−5^ s^−1^, compressive strength σ_0_ = 3.4 MPa, and elastic modulus *E*_0_ = 0.84 GPa; the dynamic enhancement coefficient CDIF of each parameter can be obtained by fitting Equations (7) and (8). Then, the dynamic size effect model given in Equation (11) was used to calculate and predict the test data of the compressive strength and elastic modulus of different specimen heights and strain rates.

**Figure 12:**
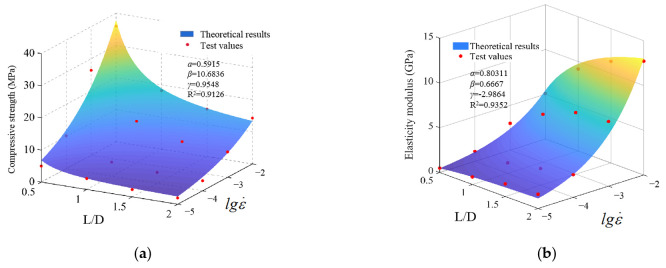
Theoretical and test values of the theoretical model of dynamic size effect. (**a**) Compressive strength. (**b**) Elastic modulus.

Figure 12 shows the predicted calculation results and test results of the modified size effect model. It is easy to know from Figure 12 that some theoretical results deviate slightly from the test values, but the variation trend of mechanical properties with height and strain rate is consistent with the analysis of the test results. The similarity between the theoretical values and experimental values preliminarily verify the rationality of the theoretical model, describing the size effect of asphalt concrete under different strain rates.

## 5. Conclusions

For studying the influence of the size effect and the strain rate effect on the mechanical properties and failure modes of RCHAC, a dynamic compression test was carried out under different strain rates. Moreover, a modified size effect theoretical model for RCHAC under different loading rates is proposed according to the influence mechanism of the strain rate and size effect. The following conclusions are obtained on the basis of the experimental studies:

(1) The strain rate and specimen size have coupling effects on the failure modes of RCHAC. When the strain rate is larger or the size is smaller, the damage degree becomes greater. When the strain rate is 10^−3^ s^−1^, the specimen with a height-to-diameter ratio of 0.5 shows shear failure. When the strain rate decreases or the size increases, the failure modes of the specimen changes gradually from shear failure to aggregate fracture and bond failure. When the strain rate is 10^−3^ s^−1^, the specimen with a height-to-diameter ratio of two has no obvious cracks, and only part of the asphalt matrix is extruded.
(2) When the ambient temperature is 5 °C, there is an obvious size effect on the mechanical properties of RCHAC when the specimen height ranges from 50 mm to 200 mm. When the specimen size increases, the compressive strength decreases non-linearly, and this decreasing trend gradually declines, while the elastic modulus increases non-linearly, and the increasing trend decreases gradually.
(3) The strain rate has an effect on the size effect of the mechanical properties of RCHAC. With the increase in strain rate, the variation trend of compressive strength and the elastic modulus with size is more significant.
(4) A dynamic size effect model considering strain rate enhancement is proposed, and the relationship between the dynamic loading rate, size, compressive strength, and elastic modulus is established, which can reasonably describe the size effect of dynamic compressive performance under strain rate effect.
(5) The proposed dynamic size effect model considering strain rate enhancement established the relationship between the dynamic loading rate, size, compressive strength, and elastic modulus, and it could reasonably describe the size effect of dynamic compressive performance under strain rates from 10^−5^ s^−1^ to 10^−2^ s^−1^.

It is to be noted that the viscosity of asphalt concrete plays an important role in its mechanical properties with the increase in specimen size at room temperature. In this paper, the combined effect of the strain rate and size on the mechanical properties and failure modes of RCHAC have been explored, but the effect of the viscosity of RCHAC was not considered. It also can be known from the failure modes and energy characteristics that with the decrease in strain rate or increase in size, the bond failure of the specimen is more significant, which also shows that viscosity has an effect on the failure mode of the specimen. Therefore, the influence of viscosity should be considered in subsequent studies on the size effect of asphalt concrete.

## Figures and Tables

**Table 1:** Compressive strength of specimens with different sizes under different strain rates (MPa).

	Strain Rate ( $\dot{\varepsilon }$ ) /s^−1^	10^−5^	10^−4^	10^−3^	10^−2^
Height/mm					
50		5.01	10.22	26.44	35.98
100		3.40	4.33	12.88	18.22
150		2.25	3.43	8.83	14.76
200		2.30	4.43	7.91	15.59

**Table 2:** Elastic modulus of specimens with different sizes under different strain rates (GPa).

	Strain Rate $\dot{\varepsilon }$ /s^−1^	10^−5^	10^−4^	10^−3^	10^−2^
Height/mm					
50		0.51	1.10	4.54	5.08
100		0.84	1.18	5.34	9.15
150		1.41	1.87	6.88	11.35
200		1.59	2.52	7.23	12.71

**Table 3:** Fitting parameters of *β* and *γ* at different strain rates.

		Strain Rate/s^−1^			
		10^−5^ s^−1^	10^−4^ s^−1^	10^−3^ s^−1^	10^−2^ s^−1^
Compressive strength	*β*_1_	10.6836	5.3997	2.3286	1.7391
	*γ*_1_	0.9548	0.8261	0.7876	0.6148
Elastic modulus	*β*_2_	0.6667	0.7931	0.8142	0.8720
	*γ*_2_	−2.9864	−2.8048	−2.4125	−2.4938

**Table 4:** Fitting parameters of *α* at different heights.

		L/D			
		0.5	1	1.5	2
Compressive strength	*α*_1_	0.6784	0.59145	0.63259	0.66595
	R^2^	0.91164	0.94579	0.98534	0.99181
Elastic modulus	*α*_2_	0.80165	0.80621	0.82631	0.74145
	R^2^	0.85927	0.95991	0.95144	0.85222

## Data Availability

Data are contained within the article.

## Nomenclature

**Symbol**	**Paraphrase**
*U*	Total input strain energy
*U* ^d^	Dissipative strain energy
*U* ^e^	Elastic strain energy
*ε*_p_	Peak strain
*σ*_p_	Compressive strength
*ε* ^e^	Elastic strain
*D*	Diameter of the specimen
f _t_	Strength of the specimen with a height of 100 mm
*γ*	The ratio of height to diameter
*F*_c_	The mechanical parameter
*P*_d_	Dynamic mechanical parameters
*P*_s_	Quasi-static mechanical parameters
${\dot{\varepsilon }}_{d}$	Dynamic strain rate
${\dot{\varepsilon }}_{s}$	Quasi-static strain rate
*D*_0_, *β*_c_, *γ*_c_, and *α*	The fitting parameters
